# Comparative Evaluation of Bioactive Compounds and Volatile Profile of White Cabbages

**DOI:** 10.3390/molecules25163696

**Published:** 2020-08-13

**Authors:** Ante Lončarić, Tihana Marček, Domagoj Šubarić, Antun Jozinović, Jurislav Babić, Borislav Miličević, Karmen Sinković, Drago Šubarić, Đurđica Ačkar

**Affiliations:** 1 Faculty of Food Technology Osijek, Josip Juraj Strossmayer University of Osijek, Franje Kuhača 20, 31000 Osijek, Croatia; tihana.marcek@ptfos.hr (T.M.); Antun.jozinovic@ptfos.hr (A.J.); jurislav.babic@ptfos.hr (J.B.); borislav.milicevic@ptfos.hr (B.M.); drago.subaric@ptfos.hr (D.Š.); dackar@ptfos.hr (Đ.A.); 2 Faculty of Agrobiotechnical Sciences Osijek, Josip Juraj Strossmayer University of Osijek, Vladimira Preloga 1, 31000 Osijek, Croatia; domagoj.subaric@fazos.hr (D.Š.); karmen.sinkovic@zg.t-com.hr (K.S.); 3 Polytechnic in Požega, Vukovarska 17, HR 34000 Pozega, Croatia

**Keywords:** physicochemical composition, phenolic acids, volatile profile, allyl isothiocyanate

## Abstract

Cabbage is an important source of bioactive compound, which is available throughout the year. However, a lot of different traditional, and hybrid varieties with different levels and composition of bioactive compounds can be found on the market. The aim of the study was to obtain quantitative results showing comparative differences between different white cabbages (“*Čepinski*”, “*Varaždinski*”, “*Bravo*”, “*Ogulinski*”) from Croatia. Morphometric parameters and physicochemical composition were determined while using standard procedures. Phenolic acids were determined using high-performance liquid chromatography and volatile compounds were analysed by the solid-phase micro-extraction gas chromatography with mass spectrometry (SPME-GC-MS) smethod. The results showed that studied cabbage cultivars differed in physicochemical composition and morphological traits. Six phenolic acids were identified and quantified, whereas a sinapic acid was the most dominant component (65.9–78.15 mg/kg). Aldehydes, esters, alcohols, and terpenes were the major classes of organic volatile compounds present in the studied cabbages. “*Čepinski*”, which has never been analysed before, showed to contain the highest amount of d-limone (40.75 µg/L) and allyl isothiocyanate (1090.26 µg/L), the most important volatile compounds responsible for the fresh cabbage flavour. The presented results mark off “*Čepinski*” cultivar as valuable for larger production and further examination.

## 1. Introduction

Cabbage (*Brassica oleracea* L.) is a widely cultivated and preferred dietary vegetable that can be consumed in various culinary forms (fresh, fermented, baked, etc.). This preference is based on its low cost and availability in local markets. In order to achieve higher yield, compacted head, uniform quality, and resistance to diseases, farmers often cultivate hybrid cabbage cultivars. The most prominent hybrid cultivar in Croatia is “*Bravo*”, since it has very good field performance and achieves high yields. However, traditional cultivars are still highly valued, because of their taste, tradition, and suitability for fermentation. In Croatia, there are several traditional cabbage cultivars, cultivated in different locations, “*Varaždinski*” in Varaždin County, “*Ogulinski*” in Karlovac County, and “*Čepinski*” in Osijek-Baranja County. Among these, “*Varaždinski*” and “*Ogulinski*” cabbages are protected in fermented form by designations of origin and geographical indications (EU) 2015/1413; (EU) 2017/1900, respectively. “*Čepinski*” cabbage is cultivated in the geographical area of Čepin (45°31′25.00′′ N 18°33′47.99′′ E) and Antunovac (45°29′14.39′′ N 18°40′28.79′′ E), and it is recognized as a guarded variety by the decision of the Ministry of Agriculture.

In addition to economical and traditional importance, cabbage has high amounts of phytochemicals and phytonutrients [1]. The studies have shown that white cabbage (*Brassica oleracea* var. *capitate f. alba*) is rich in phenolic acids and flavonols [2,3,4,5,6,7]. A previous study on polyphenol content and antiradical activity that was conducted by [8] on these four cabbage cultivars (“*Varaždinski*”, “*Ogulinski*”, “*Čepinski*” and “*Bravo*”) showed that the most characteristic phenolic acid was sinapic acid, followed by ferulic, caffeic, and *p*-coumaric acids. It is concluded that, between the studied cultivars, [8] “*Varaždinski*” had the highest polyphenol content, followed by “*Čepinski*” and “*Ogulinski*”, and the lowest amount had “*Bravo*” cultivar. Beside polyphenols, cabbage contains many flavour compounds, among which allyl isothiocyanate is the most important volatile compound for cabbage, as it gives it a characteristic, desirable aroma, and is responsible for the fresh cabbage flavour [9,10,11]. However, there are very few publications dealing with physicochemical composition, especially with in-depth analytical study of cabbage volatiles of different cabbage cultivars, traditional vs. hybrid [11,12].

The main objective of the study was to present differences between traditional and hybrid cabbage cultivars in physicochemical, bioactive, and volatile composition.

## 2. Results and Discussion

### 2.1. Physical and Morphological Properties of Studied Cabbage Cultivars

Table 1 shows a great range of morphological and physiological variations of selected cultivars. The results showed that the “*Bravo*” cultivar had the largest head mass (1677.73 g) and “*Ogulinski*” had the smallest (1384.12 g). The value of the height/width ratio indicate that “*Čepinski*” and “*Bravo*” cabbages had almost regular round shaped heads (1 and 0.85, respectively), while “*Varaždinski*” and “*Ogulinski*” had distinctively oval-shaped heads (0.67 and 0.62, respectively), which is in agreement with the results that were published by [8]. “*Čepinski*” cabbage (5.30 cm) had the shortest, while “*Varaždinski*” had the longest (8.30 cm) stem base (Table 1 and Table 2). Shorter stem base affects the distance between leaves providing the formation of solid cabbage heads [13]. Moreover, the distance from the top of the stem base to the top of the head was significantly longer in “*Čepinski*” and “*Bravo*” in regard to other cultivars, making head more compact, but firm. Such internal head organisation provides optimal conditions for anaerobic fermentation. Additionally, from the commercial point of view, a short stem base of a cabbage is a market desirable feature [14]. Higher density and height of the head has already been described in Serbian traditional cabbage cultivar “*Futoški*” [15].

The “*Čepinski*” cultivar showed the narrowest head compared to other cultivars (Table 1). “*Varaždinski*” and “*Ogulinski*” cultivars had the longest base stem and the shortest distance from the base stem to the top of the cabbage, while the head of the “*Čepinski*” and “*Bravo*” cultivars were taller than the “*Varaždinski*” and “*Ogulinski*” cultivar. Higher heads showed to be a desirable cabbage trait in providing the plant resistance against diseases, because it reduces the possibility of infection with soil pathogens [14].

Textural properties of cabbage leaves for the analysed cabbage cultivars showed that “*Ogulinski*” had the highest bio-yield point (1308.65 g), followed by “*Čepinski*” (1105.23 g), “*Varaždinski*” (833.62 g), and then “*Bravo*” (557.24 g). The harder leaves of “*Bravo*” and “*Varaždinski*” cabbage cultivars (432.85 and 430.57 g, respectively) as compared to “*Ogulinski*” and “*Čepinski*” (404.83 and 369.71 g, respectively). The visual observations also showed a stronger development of leaf nervature in “*Čepinski*” and “*Ogulinski*” than in other two cultivars (Table S1: Visual observation of cabbage leaves). All of this could be related to higher contents of insoluble fibres in “*Bravo*” and “*Varaždinski*” cabbage cultivars (2.06 and 2.12 g). Insoluble fibres generally include plant cellulose and hemicellulose, which essentially give the plant its strength. Regarding the leaf’s elasticity, “*Bravo*” cabbage cultivar showed to have more elastic leaves (88.57 mm) in comparison to other studied cabbage cultivars (Table 1 and Table 2). Colour parameters of all four studied cabbage cultivars indicate that the cabbage surface was lighter green, L parameter was above 50, a* parameter was negative, and the coordinate b* indicates a slight yellow colour. The surface of studied cabbages was greener than the surface of the cabbages after removing two leaves. This was confirmed by the greenness level parameter. However, the difference between the green colour of the surface and the surface after removing two leaves is a smaller in “*Čepinski*” and “*Ogulinski*” cultivars as compared to the “*Bravo*” and “*Varaždinski*” cultivars.

**Figure 1:**
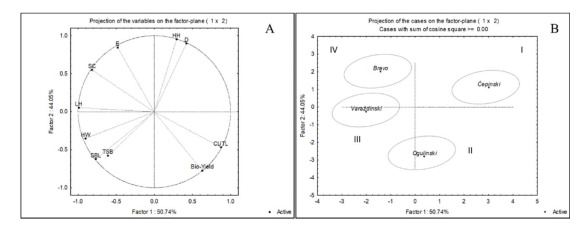
Principal component analysis of studied cabbage cultivars using morphological and physical traits. Factor loadings (**A**) and scores (**B**) of first two factors. Head hight (HH), head width (HW), distance from the top of steam base to the top of the head (D), steam base length (SBL), thickness of steam base (TSB), Bio-Yield point (Bio-Yield), leaves hardness (LH), elasticity (E), surface colour greenness (SC), and colour underneath two leaves greenness (CUTL).

Principal component analyses (PCA) was performed in order to differentiate morphological and physical traits between cultivars. Two PCA components (PC1 and PC2) contribute the most to explaining the total variance (94.78%) (Figure 1a). PC1 was mostly determined by high negative loadings on head width, stem base length, strength of the leaves, and surface colour greenness, while the colour underneath the two leaves greenness had positive loadings (Table S2: Factor loadings of morphometric parameters and physical properties). PC2 exhibited strong negative loadings on Bio-Yield point, but positive loadings on head hight, distance from the top of stem base to the top of the head, and elasticity. The results of the PCA analysis show four clusters (Figure 1b). Cluster I refers to “*Čepinski*” cultivar with lower head width (HW), stem base length (SBL), Bio-yield point, elasticity (E), and leaf hardness (LH) than “*Bravo*”. “*Ogulinski*” cabbage belongs to cluster II, because of smaller heads (HW and HH), shorter stem base length, and distance from the top of the stem base to the top of the head (D). Cluster III refers to “*Varaždinski*” cultivar due to higher head width and stem base length than “*Bravo*”, but it has lower elasticity values. Cluster IV separates “*Bravo*” mostly in terms of greater strength of the leaves and higher elasticity than “*Čepinski*”.

### 2.2. Chemical Properties of Studied Cabbage Cultivars

Table 2 shows the chemical properties of studied cabbage cultivars. The main component of all studied cabbage cultivars was water. The water content of studied cabbages varied between 90.41–93.73%, which is also reported for other white cabbage cultivars [15,16]. “*Varaždinski*” cabbage showed lower water content when compared to other studied cultivars. However, the water content of “*Varaždinski*” cabbage was similar to water content (90.34%) reported in white cabbage by [17]. Ref. [18,19] reported slightly lower water content (82.7% to 88.4%) in white cabbage. White cabbage contains relatively high levels of protein and, in this study, they ranged from 1.52–2.42 g/100 g. There were significant differences (*p* < 0.01) between the studied cabbage cultivars. The highest protein content was found in the “*Varaždinski*” cultivar and the lowest in the “*Bravo*” cultivar. These data are consistent with the results that were reported by the [20,21] for the *Brassica* cultivars. When considering ash content, it depends on the cabbage cultivar. The highest ash content was found in the “*Varaždinski*” cultivar and the lowest in the “*Ogulinski*”. However, all studied cabbage cultivars had lower ash content when compared to the other studies [20,21,22]. White cabbages (*Brassica oleracea var. capitata*) are a good source of dietary fibers [23]. “*Varaždinski*” cultivar had the highest content of insoluble fibers r (2.12%), followed by “*Bravo*” (2.06%), “*Ogulinski*” (1.87%), and “*Čepinski*” (1.62%). One of the basic parameters used in evaluating quality attributes of vegetables is sugar content and total soluble solids (TSS), which can be used as an estimate of sugar content. The results that are presented in Table 2 show correlation between total sugar content and TSS. The total sugar content in the studied cabbages varied between 3.53 and 4.23 g/100 g, regarding TSS it varied between 5.70 and 8.50 °Brix. There were significant differences (*p* < 0.01) in the total sugar and soluble solid contents among different cultivars. Furthermore, the studied cultivars had similar pH values (5.87–6.25) with the lowest pH measured in the “*Varaždinski*” cultivar. Ref. [24] reported similar pH values in their research. Higher pH values were reported for B. *oleracea* (6.06–6.44), Chinese cabbage (6.48) and B. *oleracea* L. var. *italic* (6.50) [25,26,27]. Table 2 shows the total polyphenol content in studied cabbage cultivars. The amount of TPC (from 401 to 595 mg (GAE)/kg FW) is in accordance with the other studies, where 24–856 mg GAE/kg FW were reported [28,29,30]. The TPC results revealed significant differences (*p* < 0.01) between four cabbage cultivars. “*Varaždinski*” cultivar had the highest TPC, followed by “*Čepinski*”, “*Ogulinski*” and “*Bravo*”. The presented results are in accordance with those published by [8]. Furthermore, the results for total polyphenols and antioxidant activity showed good correlation (r^2^ = 0.89).

Antioxidant activity was determined with the DPPH, as shown in Table 2. A lot of previous studies showed the ability of polyphenols found in cabbage to scavenge free radicals [2,31,32,33,34,35]. “*Čepinski*” cultivar had the highest level of antioxidant activity (1.18 µmoL DPPH/g), however not significantly different (*p* < 0.01) to “*Varaždinski*” cultivar (1.16 µmoL DPPH/g). Cabbage is considered to be a good source of vitamin C, the major antioxidants in *Brassica* spp. along phenolic compounds. Together, these components represent 80% of the total antioxidant capacity of the plants [36]. In this study, the vitamin C content ranged between 13.45–18.52 mg/100 g, with “*Čepinski*” cultivar having the highest vitamin C content (18.52 mg/100 g). The reported results of vitamin C content are in accordance with the results of a study conducted by [37] on different cabbage cultivars. In their study, vitamin C content ranged between 5.66 and 23.50 mg/100 g).

### 2.3. Phenolic Acid Content of Studied Cabbage Cultivars

Flavonoids (mainly flavonols) and the hydroxycinnamic acids are the most widespread and diverse group of polyphenols in *Brassica* species [38]. However, polyphenol profile and polyphenol content could be affected by genetic background and environmental factors, such as the season of planting, the environmental conditions, etc. [34,35]. In this study, six phenolic acids were identified and quantified (Table 3). The sinapinic acid was the major phenolic compound and it ranged from 78.15 mg/kg in “*Čepinski*” cultivar to 65.9 mg/kg in “*Ogulinski*” cultivar. Other phenolic acids were present in lower quantities of ferulic acid (4.96 to 7.77 mg/kg), 3,5-dihydroxy-benzoic acid (3.64 to 5.74 mg/kg), caffeic acid (1.49 to 2.16 mg/kg), *p*-coumaric acid (0.41 to 1.27 mg/kg), and chlorogenic acid (0.24 to 0.43 mg/kg). Ref. [22] reported a little bit higher contents of caffeic acid (2.5 to 3.1 mg/kg FW) and *p*-coumaric acid (1.2 to 1.5 mg/kg FW) when compared with our study. Ref. [12] found an average of 1.56 mg/kg FW of caffeic acid, 0.90 mg/kg FW of *p*-coumaric acid, and 0.97 mg/kg FW of ferulic acid studying different cabbage cultivars. In our study, “*Varaždinski*” cabbage had significantly (*p* < 0.01) higher amounts of all individual polyphenols when compared to other studied cabbages. Furthermore, it could be noticed that “*Čepinski*” and “*Varaždinski*” cabbages are distinguished from other cultivars by the high amount of sinapic acid. Other authors reported sinapic acid as the major phenolic acid, whereas caffeic acid, *p*-coumaric acid, and ferulic acids have also been identified in white cabbages [39,40].

### 2.4. Volatile Profile of Studied Cabbage Cultivars

The numerous volatile compounds that are present in fresh cabbages are dominated by aldehydes and ketones (20), terpens (10), alcohols (8), esters (10), acids (3), and miscellaneous compounds (4) when extracted using HS-SPME and analysed by the GC-MS (Table 4). “*Varaždinski*” cultivar had the highest content of aldehydes and ketones (4478.59 µg/L), followed by “*Čepinski*” (2953.99 µg/L). “*Bravo*” and “*Ogulinski*” both had lower aldehydes and ketones content without significant difference (*p* < 0.01) between them. The most prominent volatiles from the aldehyde series were 2-hexenal, nonanal and 2-octanal. 2-hexenal and nonanal are both characterized by a citrus and green odour, while 2-octanal is described as having a nutty flavour. The highest content of 2-hexanal had “*Varaždinski*” followed by “*Čepinski*”, “*Ogulinski*”, and “*Bravo*” cabbage cultivars (3287.62, 2207.64, 1035.35, 948.58 µg/L, respectively). “*Čepinski*” cultivar had lower content of nonanal and 2-octanal when compared to other investigated cultivars. Regarding the ketones, “*Čepinski*” had the highest content of geranyl acetone (102.23 µg/L), a monoterpene ketone that is characterized by a leaf floral green odour. “*Bravo*” and “*Varaždinski*” had 90.18 and 80.17 µg/L, while, in it, was not detected “*Ogulinski*”.

A total of ten terpenes were detected in the studied cabbage cultivars. Estragol, describing spice green herbal odour, was unique in “*Čepinski*” and “*Varaždinski*” cultivar, while elemicin, describing spice flower odour, in “*Bravo*” and “*Ogulinski*”. The most dominant terpenes detected were carveol (30.48–72.99 µg/L) and miristicine (36.60–75.79 µg/L). Interestingly “*Čepinski*” cultivar had by far the larger content of d-limonen (40.75 µg/L), which gives the characteristic green aroma of cabbages, as reported by [9,41]. The composition of alcohols contributing to the flavour of cabbages is not described in detail so far. It can be seen from the results that “*Čepinski*” and “*Varaždinski*” cultivars are dominated by 2-ethyl hexanol (89.83 and 77.24 µg/L, respectively) and tetradecanol (88.45 and 30.63 µg/L, respectively). “*Bravo*” cultivar is dominated by tetradecanol (56.11 µg/L) and perilyl alcohol (50.56 µg/L), while “*Ogulinski*” with 2-ethyl hexanol (45.91 µg/L) and perillyl alcohol (118.06 µg/L). The named alcohols, 2-ethyl hexanol, tetradecanol, and perillyl alcohol, are described by citrus, fruity, and green odours contributing to the characteristic fresh cabbage aroma.

We can see in Table 4 that several esters in the form of isothiocyanates (five compounds) were found in studied cabbage cultivars. Several isothiocyanates were also identified and reported to be present in fresh cabbages by the [42]. Isothiocyanates are among the major components that are responsible for the pungent aroma of cabbage and these are produced by enzymatic conversion of metabolites, called glucosinolates, a sulphur group that contains amino-acid derived compounds [10]. Phenyl ethyl isothiocyanate was the most dominant volatile in “*Bravo*”, “*Čepinski*”, and “*Varaždinski*” cultivar (1382.36, 1056.69 and 1050.93 µg/L, respectively). The highest content of benzyl isothiocyanate was detected in ‘*Ogulinski*’ (1507.64 µg/L), followed by “*Varaždinski*” (868.10 µg/L) and “*Bravo*” (403.96 µg/L). Allyl isothiocyanate is considered to be the most important volatile compound that is responsible for the fresh cabbage flavour [9]. The highest content (1009.26 µg/L) of allyl isothiocyanate was detected in the “*Čepinski*” cultivar. Other studied cultivars had a significantly smaller content of allyl isothiocyanate (66.55–311.44 µg/L).

Diisobutyl phthalate was fourth odor detected in higher content in all studied cabbage cultivars in the range of 126.49–393.85 µg/L. Regarding the miscellaneous compounds the most prominent odour was 2,4-di-t-butylphenol with the ranges between 405.05 and 754.28 µg/L. Although the volatile compounds of cabbages in this study closely followed several earlier reports this work reports for the first time a detailed volatiles profile of cabbages commonly grown in Croatia.

## 3. Materials and Methods

### 3.1. Chemicals

Chlorogenic acid (C3878, ≥ 95%), caffeic acid (C0625, 99%), ferulic acid (F3500, 99%), *p*-coumaric acid (C9008, ≥ 98%), sinapic acid (D7927, ≥ 98%), 3,5-dihydroxybenzoic acid (D10,000, 97%), and 2,2-diphenyl-1-picryl-hydrazyl radical (DPPH, D9132) were purchased from Sigma–Aldrich (St. Louis, MO, USA). Orto-phosphoric acid 85%, HPLC grade, methanol HPLC grade, hydrochloric acid 36.2% were purchased from Fluka (Buchs, Switzerland) and Folin–Ciocalteu reagent and sodium carbonate from Kemika (Zagreb, Croatia).

### 3.2. Cabbages Used for the Experiment

Freshly harvested white cabbages (*Brassica oleracea* var. *capitate f. alba*) from Croatia were studied—‘*Čepinski*’, ‘*Varaždinski*’, ‘*Bravo*’, and ‘*Ogulinski*’. The protocol described by [8] was followed in order to prepare samples for the analysis. Each cabbage cultivar consisted of ten cabbages, which were harvested from local producers in October 2019. Briefly, the cabbages were equally cut into three pieces and the core was removed. The homogenous samples were obtained by pooling together pieces, one from each cabbage. These homogenous samples were ground with the use of a stick blender. The extracts were prepared immediately after the homogenization. The extracts for determination of total polyphenols, antioxidant activity (DPPH) and individual polyphenols with HPLC-PDA were obtained by extracting 0.2 g of cabbage in 80% methanol as a solvent. Extraction was performed in ultrasonic bath RK 100 (Bandelin Sonorex, Berlin, Germany) for 15 min., and centrifuged for 10 min. at 10,000 rpm (Minispin Eppendorf, Germany). The extracts were separated from the residues and then the residues were extracted two more times with the same solvent. Extracts were combined.

Figure S1 shows all of the cabbages used in the experiment: Cabbage cultivars used in experiment.

### 3.3. Physical Analysis

Raw cabbage head weight was measured by scale balance (Kern, UK) and the width of raw cabbage was measured with the ruler at the site of widest cabbage cross-section. The height of cabbage head was measured in the middle of the cross section from the stem base to the top of the cabbage head. Stem length of cabbage heads were also measured. All of the measurements were done on ten cabbage heads and descriptive statistics are presented in following tables.

### 3.4. Texture Analysis

The texture properties of fresh cabbages were evaluated using a texturometer TA-XT2 (Stable Micro Systems, Godalming, UK) following the procedure that was described by [15]. Briefly, the test settings were: test speed 1.0 mm/s, distance 10 mm, trigger force 5 g. The exact punch position was set on left-hand side of the midway between leaf tip and base, between the midrib and the leaf margin, avoiding the main veins. Mean burst strength and mean distance to burst were determined as indicators of leaves hardness and elasticity. All of the measurements were done on ten cabbages.

### 3.5. Colour Measurement

Colour was measured using a colorimeter (Konica Minolta CR-10, Osaka, Japan) after calibration against a ceramic reference. The following parameters were obtained; L* (brightness), a* (redness), b* (blueness). The colour was measured on surface of cabbage head and after removing first two outer leaf layers from the cabbage head. The green-ness was calculated from the ratio -a*/b*, as previously reported by [43].

### 3.6. Morphological Analysis

**Figure 2:**
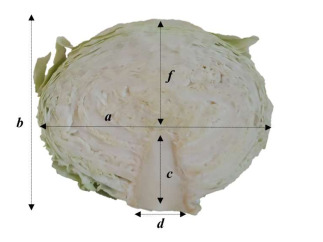
Morphological determination marks. Head width (**a**), head height (**b**), stem base length (**c**), thickness of steam base, (**d**) and distance from the top of stem base to the top of the head (**f**).

Three representative heads of each cultivar with marketable size were used for the morphological evaluation. The heads were cut into two parts and the measurements were performed while using the ruler according scheme listed below (Figure 2).

### 3.7. Chemical Analysis

The moisture percentage in cabbage samples was measured using the ISO 6540 method, crude protein content ISO 5983-2, and crude ash ISO 5984 method. Total, soluble, and insoluble dietary fibres were measured using AOAC 991.43. The content of soluble solids of cabbages was measured with a tabletop Abbe refractometer and given in Brix (°Brix). The reducing and total sugars were determined by Luff Schoorl’s method [44].

### 3.8. Total Polyphenol Determination

The total polyphenol content (TPC) was determined by using the Folin–Ciocalteu method (FC), according to a procedure described by [45]. The changes in the colour of the radical from light blue to dark blue were measured after 30 min. at 760 nm, using a UV-ViS spectrophotometer (Shimadzu UV-1800, Kyoto, Japan). Gallic acid was used as a standard and the total polyphenols were expressed as mg of gallic acid equivalents (GAE) per kg of fresh weight of cabbage from the calibration curve (50–600 mg/kg, r^2^ = 0.9984) while using gallic acid.

### 3.9. Antioxidant Activity Measured with DPPH Method

The antioxidant activity of cabbage extracts was determined by 1.1-diphenyl-1-picrylhydrazyl (DPPH) method [46]. For this purpose, 0.2 mL of cabbage extracts were mixed with 3.8 mL of a DPPH radical solution 0.1 mM in methanol and shaken well. Those samples were kept 30 min. in the dark place at 25 °C. Absorbance was then at 515 nm using a UV-ViS spectrophotometer (Shimadzu UV-1800, Kyoto, Japan). All of the analyses were carried out in triplicate. The activity was calculated using the following equation (y = 0.9554x + 0.0282; r^2^ = 0.9947), obtained from linear regression after plotting the A_515nm_ of known solutions of trolox against the concentration (0.1–0.9 mM). The results were expressed as millimoles of Trolox^®^ equivalents (TE) per one gram of dry apple peel (mmol TE/g dw).

### 3.10. Vitamin C Method

Square-wave voltammetry was conducted at potentiostat/galvanostat Autolab while using GPES version 4.9.005 software (Eco Chemie B.V., Utrecht, The Netherlands). The electrochemical measurement was done in electrochemical cell where glassy carbon was working electrode, Pt wire counter and Ag/AgCl reference electrode. Carrier of electrolyte was 0.1 M KCl. The parameters were as follows: start potential −0.1 V, final potential 1 V, step potential 0.002 V, amplitude: 0.05 V, and frequency 50 Hz. All of the samples were tested in triplets.

### 3.11. Volatile Extraction and Analysis

The cabbage volatile profile was determined by gas chromatography with mass spectrometry (GC-MS) by a solid-phase micro-extraction (SPME) method. The extraction method was based on modified procedure that was described by [16]. Briefly, the samples were prepared by stirring 5 mL of sample and 1 g of sodium chloride in a sealed vial. After preheating (5 min. at 40 °C) volatiles were collected by SPME for 20 min. at 40 °C using 65 μm PDMS/DVB fibre (Supelco Inc., Bellefonte, PA, USA). An analysis was carried out with gas chromatography Agilent 5890 B with a mass detector Agilent 5977 A. The capillary column used in this experiment was CP-WAX52CB (Agilent, 60 m × 250 μm × 0.25 μm). Helium (He) 5.0 (purity 99.999%; Messer, Austria) was used as a carrier gas. Working conditions were as follows: injector temperature at 250 °C; MSD interface temperature 250 °C; oven temperature programmed from 40 °C (2 min. hold) to 230 °C (5 min, hold) at 6 °C/min; carrier gas (He) at a flow rate of 1 mL/min. (average velocity 25.502 cm/sec); injection port operated in split less mode. The compounds were identified by comparing their mass spectra with the spectral library (Wiley 9, NIST 0.8) and expressed as peak area. Three replicate measurements were performed for each sample.

### 3.12. Statistical Analyses

Statistical analyse of certain morphological trait included the one-way ANOVA analysis of variance and Fisher’s post hoc test to find statistical significance between cultivars. Principal component analysis (PCA) was used to discriminate the morphological traits of cabbage cultivars. Ten variables were included in PCA analysis. Loading values that were higher than >0.75 were considered as strong correlation [47]. The data were analysed with Statistica 13.5 (TIBCO Software Inc., Palo Alto, CA, USA).

## 4. Conclusions

In this study, morphometric parameters, physicochemical composition, polyphenol content, antiradical activity, and volatile profile of four cabbage cultivars “*Čepinski*”, “*Bravo*”, “*Varaždinski*” and “*Ogulinski*” were studied. Overall results of physicochemical composition and polyphenol content showed that “*Čepinski*” usually followed “*Varaždinski*” cultivar. Morphometric data showed similarities between “*Čepinski*” and “*Bravo*”, but distinguish “*Čepinski*” from “*Bravo*” due to more pronounced nervature, narrow head, and the shortest stem base making it more suitable for the fermentation process. The sinapinic acid was the major phenolic compound detected in all studied cultivar with the highest content in “*Čepinski*” cultivar. Aldehydes, esters, alcohols, and terpenes were the major classes of organic volatile compounds present in cabbage. “*Čepinski*” cultivar was never analysed before regarding the volatile profile. This study showed some interesting results showing that “*Čepinski*” cultivar contained a high amount of d-limone and allyl isothiocyanate, the most important volatile compounds responsible for the fresh cabbage flavour. The found differences in overall quality of studied cabbage cultivars could be attributed to (i) cultivars and possibly (ii) different geographical regions and climate. However, from the presented results, it is justified to consider “*Čepinski*” cultivar as valuable for bigger production and further examination.

## Figures and Tables

**Table 1:** Physical and morphological properties of cabbage cultivars.

	*‘Čepinski’*	*‘Varaždinski’*	*‘Bravo’*	*‘Ogulinski’*
Head mass (g)	1549.28 ± 267.47^bc^	1487.62 ± 348.77^bc^	1677.73 ± 191.17^ab^	1384.12 ± 316.44^cd^
Head width (cm)	14.70 ± 0.20^c^	18.40 ± 0.36^a^	16.63 ± 1.46^b^	16.90 ± 0.69^ab^
Head height (cm)	14.70 ± 1.59^a^	12.37 ± 1.00^bc^	14.17 ± 0.61^ab^	10.47 ± 0.65^c^
Height/width ratio	1	0.67	0.85	0.62
Stem base length (cm)	5.30 ± 0.98^c^	8.30 ± 0.61^a^	6.70 ± 0.70^b^	7.87 ± 0.32^ab^
Thickness of stem base (cm)	3.50 ± 0.10^a^	3.83 ± 0.21^a^	3.93 ± 0.51^a^	4.10 ± 0.62^a^
Distance from the top of stem base to the top of the head (cm)	8.20 ± 0.30^a^	4.70 ± 1.21^b^	7.60 ± 0.87^a^	3.80 ± 0.61^b^
Texture				
Bio-yield point (g)	1105.23 ± 8.99^b^	833.62 ± 8.99^c^	557.24 ± 9.07^d^	1308.65 ± 10.75^a^
Leaves hardness (g)	369.71 ± 22.72^b^	430.57 ± 11.74^a^	432.85 ± 16.63^a^	404.83 ± 14.26^ab^
Elasticity (mm)	69.71 ± 2.71^b^	73.57 ± 1.75^b^	88.57 ± 1.19^a^	60.84 ± 1.89^c^
Surface colour				
L	68.66 ± 5.23^a^	54.10 ± 7.23^c^	62.77 ± 8.25^ab^	72.69 ± 3.03^b^
a*	−18.78 ± 1.43^b^	−18.75 ± 2.80^ab^	−18.65 ± 1.35^b^	−21.37 ± 0.84^a^
b*	34.98 ± 0.83^b^	31.31 ± 7.36^bc^	31.12 ± 4.31^bc^	40.03 ± 1.20^a^
greenness level (-a*/b*)	0.54	0.60	0.60	0.53
Colour underneath two leaves				
L	75.77 ± 3.50^b^	77.12 ± 1.81^b^	81.23 ±1.09^a^	81.33 ± 1.38^a^
a*	−15.37 ± 2.65^ab^	−14.54 ± 1.11^b^	−12.40 ± 0.43^c^	−16.36 ± 0.97^a^
b*	29.71 ± 2.96^b^	30.83 ± 2.76^ab^	24.74 ± 0.76^c^	31.69 ± 1.72^a^
greenness level (-a*/b*)	0.52	0.50	0.50	0.52

Different letters in each row indicate significant differences at 95% confidence level as obtained by the LSD test.

**Table 2:** Chemical properties of cabbage cultivars (*‘Čepinski’*, *‘Varaždinski’*, *‘Bravo’*, *‘Ogulinski’*).

	*‘Čepinski’*	*‘Varaždinski’*	*‘Bravo’*	*‘Ogulinski’*
Water content (%)	93.10 ± 0.025^b^	90.41 ± 0.080^a^	93.61 ± 0.065^cd^	93.73 ± 0.055^d^
Crude protein (g/100g)	1.89 ± 0.025^b^	2.42 ± 0.015^a^	1.52 ± 0.025^d^	1.59 ± 0.005^c^
Crude ash (g/100g)	0.63 ± 0.010^b^	0.70 ± 0.005^a^	0.61 ± 0.005^c^	0.53 ± 0.010^d^
Insoluble fibres %	1.62 ± 0.002^d^	2.12 ± 0.005^a^	2.06 ± 0.003^b^	1.87 ± 0.001^c^
Soluble fibres %	0.74 ± 0.001^b^	0.43 ± 0.000^d^	0.83 ±0.001^a^	0.67 ± 0.002^c^
Total fibres %	2.35 ± 0.007^d^	2.55 ± 0.001^b^	2.89 ± 0.003^a^	2.54 ±0.000^c^
Invert sugars (g/100 g)	3.64 ± 0.002^b^	3.65 ± 0.002^a^	3.48 ± 0.004^c^	2.97 ± 0.001^d^
Total sugars (g/100 g)	3.96 ± 0.001^b^	4.23 ± 0.002^a^	3.62 ± 0.000^c^	3.53 ± 0.001^d^
Soluble solids content (°Brix)	6.90 ± 0.02^b^	8.50 ± 0.01^a^	6.70 ± 0.01^c^	5.70 ± 0.01^d^
pH	6.11 ± 0.13^a^	5.87 ± 0.07^b^	6.25 ± 0.06^a^	6.16 ± 0.09^a^
TPC (mg (GAE)/kg FW)	496.02 ± 5.88^b^	594.80 ± 7.43^a^	401.62 ± 11.10^d^	480.95 ± 1.40^c^
DPPH (µmoL DPPH/g sample)	1.18 ± 0.008^a^	1.16 ± 0.057^ab^	0.71 ± 0.003^d^	1.02 ± 0.001^c^
Ascorbic acid (mg/100 g)	18.52 ± 0.64^a^	15.19 ± 0.97^b^	13.45 ± 0.49^d^	14.35 ± 0.66^c^

Different letters in each row indicate significant differences at 95% confidence level as obtained by the LSD test.

**Table 3:** Phenolic acid content (mg/kg) of cabbage cultivars (*‘Čepinski’*, *Varaždinski’*, *‘Bravo’*, *‘Ogulinski’*).

	*‘Čepinski’*	*‘Varaždinski’*	*‘Bravo’*	*‘Ogulinski’*
Caffeic acid	1.76 ± 0.10^b^	2.06 ± 0.16^a^	1.49 ± 0.04^c^	2.16 ± 0.08^a^
*p*-coumaric acid	0.64 ± 0.06^c^	1.27 ± 0.04^a^	0.41 ± 0.03^d^	0.66 ± 0.02^c^
Ferulic acid	6.52 ± 0.15^d^	8.18 ± 0.09^a^	4.96 ± 0.17^e^	7.77 ± 0.013^b^
Sinapic acid	78.15 ± 0.28^b^	96.45 ± 0.18^a^	68.56 ± 0.16^c^	65.9 ± 0.22^d^
Chlorogenic acid	0.24 ± 0.05^c^	0.43 ± 0.06^a^	0.33 ± 0.01^b^	0.25 ± 0.04^c^
3,5-Dihydroxy-benzoic acid	4.11 ± 0.13^c^	5.74 ± 0.08^a^	3.64 ± 0.06^d^	4.61 ± 0.08^b^

Values are mean ± SD of three extracts each measured once. LOD and LOQ as follows: caffeic acid 0.15, 0.70; *p*-coumaric acid 0.09, 0.21; ferulic acid 0.20, 0.33; sinapic acid 0.11, 0.37; chlorogenic acid 0.09, 0.12 and 3, 5-dihydroxy- benzoic acid 0.04, 0.19 mg/kg. Coefficient of variation from 0.9 to 25%. FW ± fresh weight; * Different letters in each row indicate significant differences at 95% confidence level as obtained by the LSD test.

**Table 4:** Aromatic profile (µg/L) of cabbage cultivars (*‘Čepinski’*, *‘Varaždinski’*, *‘Bravo’*, *‘Ogulinski’*).

Compound	RT	RI	*‘Čepinski’*	*‘Varaždinski’*	*‘Bravo’*	*‘Ogulinski’*
**Aldehydes and Ketones**			2953.99 ± 56.23	4478.59 ± 181.72	1883.3 ± 19.68	1839.62 ± 136.91
Hexanal	13.0730	1078	98.99 ± 0.1	71.41 ± 1.16	100.62 ± 1.05	136.41 ± 2.53
Cis-3-hexenal	15.4762	1151	n.d.	n.d.	8.11 ± 0.07	n.d.
2-hexenal	17.6132	1215	2207.64 ± 34.96	3287.63 ± 50.67	948.58 ± 3.75	1035.35 ± 98.32
Octanal	19.1960	1268	20.15 ± 13.68	18.41 ± 1.07	7.5 ± 0.04	7.05 ± 0.43
2-heptenal	20.8480	1322	44.93 ± 0.46	19.45 ± 0.2	30.88 ± 0.17	36.74 ± 1.99
6-methyl-5-heptene-2-one	21.1484	1333	13.69 ± 0.32	n.d.	11.76 ± 0.01	4.05 ± 0.1
Nonanal	22.7485	1388	112.82 ± 2.45	208.46 ± 4	176.75 ± 6.83	124.24 ± 12.33
2,4-heksadienal	23.1586	1402	27.72 ± 0.04	167.51 ± 9.43	61.24 ± 0.45	75.46 ± 5.63
2-octanal	23.8287	1428	73.86 ± 0.58	310.61 ± 11.87	229.82 ± 3.2	201.14 ± 11.62
2,4-heptadienal	24.7991	1464	43.01 ± 0.12	32.23 ± 0.63	30.78 ± 0.19	35.66 ± 0.1
Benzaldehyde	26.4743	1528	17.67 ± 0.34	22.75 ± 0.85	23.39 ± 0.13	27.85 ± 0.56
2-nonenal	26.5956	1533	24.55 ± 0.25	43.21 ± 0.39	30.55 ± 0.23	34.13 ± 0.89
3,5-octadiene-2-one	27.5025	1569	31.08 ± 0.02	59.58 ± 1.1	33.56 ± 1.31	33.25 ± 0.62
2,6-nonadienal	27.8895	1584	22.85 ± 0.2	14.57 ± 0.49	12.61 ± 0.52	16.46 ± 0.14
Undecanal	28.2188	1597	34.86 ± 0.02	n.d.	23.27 ± 0.13	n.d.
2-decenal	29.2470	1641	11.76 ± 0.18	23.3 ± 0	9.1 ± 0.02	18.14 ± 0.03
4-ethyl benzaldehyde	30.9684	1714	29.9 ± 0.82	61.07 ± 50.69	22.82 ± 0.35	13.01 ± 0.78
2 4-decadienal	32.0774	1763	28.99 ± 0.3	58.24 ± 47.46	14.61 ± 0.35	33.06 ± 0.81
Geranyl acetone	33.9201	1847	102.23 ± 1.15	80.17 ± 1.71	90.18 ± 0.59	n.d.
Tetradecanal	35.3527	1914	7.29 ± 0.24	n.d.	17.17 ± 0.29	7.62 ± 0.04
**Terpens**			243.34 ± 1.88	217.19 ± 7.05	241.36 ± 1.19	165.87 ± 7.37
Verbenen	14.3033	1113	23.76 ± 0.07	12.46 ± 0.36	6.66 ± 0.07	11.18 ± 0.51
D-limonene	16.7987	1189	40.75 ± 0.22	26.11 ± 1.24	14.24 ± 0.02	21.77 ± 0.6
β-cyclocitral	28.8637	1625	13.74 ± 0.1	8.72 ± 0.18	8.48 ± 0.09	8.59 ± 0.46
Estragole	29.9343	1670	14.48 ± 0.31	8.7 ± 0.04	n.d.	n.d.
Felandral	31.3149	1729	28.57 ± 0.12	21.23 ± 0.02	22.68 ± 0.54	24.93 ± 0.74
Camphene	31.8233	1752	19.64 ± 0.41	12.93 ± 1.21	15.75 ± 0.13	9.19 ± 0.78
Carveol	33.5389	1829	59.7 ± 0.07	54.17 ± 0.54	72.99 ± 0.04	30.48 ± 1.49
Elemicin	41.2796	2234	n.d.	9.19 ± 0.06	n.d.	6.12 ± 0.13
Myristicin	42.1746	2276	42.7 ± 0.56	56.77 ± 3.25	75.79 ± 0.14	36.6 ± 1.7
Farnesol	42.2844	2281	n.d.	6.92 ± 0.16	24.78 ± 0.15	17.01 ± 0.96
**Alcohols**			382.85 ± 2.34	195.63 ± 5.1	255.18 ± 1.18	311.48 ± 15.6
2-Penten-1-ol	20.5708	1312	6.91 ± 0.08	12.97 ± 0.12	4.84 ± 0.09	8.81 ± 0.17
2-ethyl hexanol	25.1630	1478	89.83 ± 0.33	77.24 ± 1.72	47.32 ± 0.68	45.91 ± 1.81
Octanol	26.9017	1545	48.53 ± 0.29	21.98 ± 0.32	21.9 ± 0.05	30.84 ± 2.04
2-octenol	28.3690	1603	30.96 ± 0.01	8.48 ± 0.03	8.76 ± 0.02	9.3 ± 0.85
1-nonanol	29.3856	1647	55.68 ± 0.34	27.68 ± 0.79	33.59 ± 0.1	38.86 ± 1.04
Decanol	31.7540	1749	35.23 ± 0.36	16.67 ± 1.03	32.1 ± 0.02	40.87 ± 2.82
Tetradecanol	36.1152	1952	88.45 ± 0.56	30.63 ± 1.1	56.11 ± 0.16	18.83 ± 0.44
Perillyl alcohol	37.1667	2003	27.28 ± 0.36	n.d.	50.56 ± 0.07	118.06 ± 6.41
**Acids**			23.61 ± 0.32	57.1 ± 2.75	30.01 ± 0.34	18.53 ± 0.69
Nonanoic acid	40.1586	2173	9.24 ± 0.12	25.42 ± 0.36	n.d.	n.d.
Decanoic acid	42.0649	2271	8.61 ± 0.17	23.7 ± 2.37	15.5 ± 0.02	6.06 ± 0.14
Lauric acid	46.0102	2479	5.75 ± 0.04	7.98 ± 0.01	14.5 ± 0.31	12.47 ± 0.55
**Esters**			2758.96 ± 17.7	2860.92 ± 85.24	2191.54 ± 34.13	2247.88 ± 131.69
Isobutyl isothiocyanate	20.6978	1316	23.26 ± 0.1	27.88 ± 2.25	36.38 ± 0.37	27.52 ± 1.28
Allyl isothiocyanate	22.4019	1377	1090.26 ± 9.37	311.14 ± 14.9	66.55 ± 0.71	93.92 ± 5.27
Pentyl isothiocyanate	25.3768	1485	12.88 ± 0.35	26.32 ± 1.16	16.7 ± 0.13	5.97 ± 0.09
Isopropyl myristate	37.4784	2019	12.54 ± 0.21	59.14 ± 0.07	46.55 ± 0.37	7.73 ± 0.06
Benzyl isothiocyanate	39.2922	2116	163.21 ± 1.72	868.1 ± 1.89	403.96 ± 5.82	1507.64 ± 93.14
Phenyl ethyl isothiocyanate	41.6086	2250	1056.69 ± 2.2	1050.93 ± 19.66	1382.36 ± 22.7	408.61 ± 23.48
Methyl dihydrojasmonate	42.4461	2289	51.05 ± 0.92	24.6 ± 0.51	16.3 ± 0.08	48.68 ± 2.14
Diethyl phthalate	44.0173	2376	10.88 ± 0.46	52.63 ± 43.06	12.21 ± 0.33	5.83 ± 0.3
Diisobutyl phthalate	47.4659	2546	293.28 ± 2.21	393.85 ± 0.42	166.9 ± 3.42	126.49 ± 5.32
Dibutyl phthalate	51.7347	2708	44.92 ± 0.17	46.32 ± 1.34	43.64 ± 0.2	15.5 ± 0.63
**Miscellaneous compounds**			954.32 ± 13.7	741.33 ± 64.88	615.02 ± 6.4	859.77 ± 28.65
3-butene nitrile	16.3829	1177	30.32 ± 0.02	26.51 ± 1	23.53 ± 0.06	31.36 ± 1.62
2-pentilfuran	17.8732	1224	11.74 ± 0.05	61.37 ± 49.94	96.09 ± 0.46	83.77 ± 13.71
Phenylacetonitrile	35.8263	1938	157.98 ± 7.44	56.26 ± 0.37	32.07 ± 0.41	339.59 ± 2.82
2,4-di-t-butylphenol	42.6772	2299	754.28 ± 6.19	597.19 ± 13.58	463.33 ± 5.47	405.05 ± 10.51
* internal standard: mirtenol	32.7417	1792				

## Supplementary Materials

The following are available online, Figure S1: Cabbage cultivars used in experiment, Table S1: Visual observation of cabbage leaves, Table S2: Factor loadings of morphometric parameters and physical properties.
