# Supplementary material for: Comparative Evaluation of Bioactive Compounds and Volatile Profile of White Cabbages
Source: Molecules. 2020 Aug 13;25(16):3696. doi: 10.3390/molecules25163696 (PMC7464038; doi:10.3390/molecules25163696)
Supplement: Supplementary file 1 [file molecules-25-03696-s001.pdf]

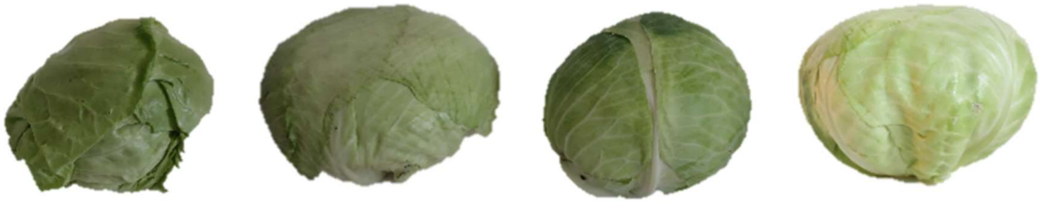

'Čepinski' *'Varaždinski'* *'Bravo'* *'Ogulinski'*

S1 Figure. Cabbage cultivars used in experiment

S2 Table. Visual observation of cabbage leaves

Cultivar	Leaf blade brittleness	Nervature hardness
'Čepinski'	++	+
'Varaždinski'	++	+++
'Bravo'	+	+++
'Ogulinski'	+++	++

The most pronounced (+++), medium pronounced (++), the less pronounced (+)

S3 Table. Factor loadings of morphometric parameters and physical properties.

Variable	PC1	PC2
HW	-0.902	-0.354
HH	0.291	0.953
SBL	-0.768	-0.624
TSB	-0.611	-0.577
D	0.422	0.899
Bio-Yield	0.630	-0.773
LH	-0.990	0.054
E	-0.482	0.845
SC	-0.816	0.551
CUTL	0.872	-0.471
Explained variance (eigenvalue)	5.07	4.40
Proportion of total variance (%)	50.74	44.05
Cumulative variance (%)	50.74	94.79

PC-1 (principal component 1); PC-2 (principal component 2); HW (head width), HH (head hight), SBL (steam base lenght), TSB (thickness of steam base), D (distance from the top of steam base to the top of the head), Bio-Yield (Bio-Yield point); LH (leaves hardness); E (elasticity); SC (Surface colour greennness); CUTL (Colour underneath two leaves greenness)
